# Leveraging the tolerogenic potential of TNF-α and regulatory B cells in organ transplantation

**DOI:** 10.3389/fimmu.2023.1173672

**Published:** 2023-04-27

**Authors:** Sonya A. Poznansky, Matthew Yu, Kevin Deng, Qiang Fu, James F. Markmann, Christian LeGuern

**Affiliations:** ^1^ Center for Transplantation Sciences, Department of Surgery, Massachusetts General Hospital, Harvard Medical School, Boston, MA, United States; ^2^ Organ Transplantation Center, Sichuan Provincial People’s Hospital and School of Medicine, University of Electronic Science and Technology of China, Chengdu, China

**Keywords:** TNF-α, Bregs-TLR, TNFR1, TNFR2, tolerance, transplantation, immunology

## Abstract

A subset of B-cells with tolerogenic functions, termed B-regulatory cells or Bregs, is characterized by the expression of anti-inflammatory/tolerogenic cytokines, namely IL-10, TGF-β, and IL-35, that contribute to their regulatory functions. Breg regulation favors graft acceptance within a tolerogenic milieu. As organ transplantation invariably triggers inflammation, new insights into the crosstalk between cytokines with dual properties and the inflamed milieu are needed to tailor their function toward tolerance. Using TNF-α as a proxy of dual-function cytokines involved in immune-related diseases and transplantation settings, the current review highlights the multifaceted role of TNF-α. It focuses on therapeutic approaches that have revealed the complexity of TNF-α properties tested in clinical settings where total TNF-α inhibition has proven ineffective and often detrimental to clinical outcomes. To improve the efficacy of current TNF-α inhibiting therapeutics, we propose a three-prong strategy to upregulate the tolerogenic pathway engaging the TNFR2 receptor while simultaneously inhibiting the inflammatory mechanisms associated with TNFR1 engagement. When combined with additional administrations of Bregs-TLR that activate Tregs, this approach may become a potential therapeutic in overcoming transplant rejection and promoting graft tolerance.

## Introduction

Although B cells may be most well-known as antibody producers, they also display a gamut of other properties, including the presentation of antigens to T cells. This function is modulated by the differential expression of costimulatory molecules, leading to the regulation of T-cell activation. Furthermore, B cells tailor their cellular environment via the secretion of cytokines and chemokines (1). In transplantation, B cells may produce graft-specific antibodies that accelerate graft destruction or contribute to immunomodulatory processes that foster graft tolerance (2). There are many known subsets of B cells defined by their different stages of maturation, activation, and function. Some B cell subsets, the B-regulatory cells or Bregs, have common regulatory properties that include the ability to inhibit T and B lymphocyte functions and, consequently, mitigate tissue inflammation and immune suppression (2).

Bregs’ immunomodulatory functions are often associated with the production of anti-inflammatory cytokines, notably interleukin-10 (IL-10) (2–4). Bregs have also been observed to release Tumor Necrosis Factor alpha (TNF-α), a cytokine with both pro- and anti-inflammatory signaling pathways (5, 6). This potentially pro-inflammatory cytokine may compromise Breg tolerogenic function via mechanisms potentially similar to those blocking Treg or conventional T cell activation (7, 8). TNF-α is thought to engage two distinct receptors on targeted cells, TNFR1 and TNFR2, and activate two opposing signaling pathways. Nearly all cell types express TNFR1, which, when bound by TNF-α, triggers inflammation mechanisms and the recruitment of proliferating immune cells. Conversely, the binding of TNF-α to the TNFR2 receptor, expressed only on immune and nervous system cells, activates homeostatic bioactivities, the induction of anti-inflammatory responses, cell regeneration, proliferation, and survival (9, 10).

Thus, depending on the local pro-tolerogenic or -inflammatory environment, the TNF-α receptor binding duality can result in tolerogenic or inflammatory responses to transplants. The potential to manipulate receptor binding has not yet been closely examined and could improve graft acceptance. Given that TLRs are recruited during graft reperfusion and transplantation, it appears essential to understand the modalities of preserving a pro-tolerogenic graft milieu favorable to immune regulation via Bregs and other regulatory cells and cytokines. Furthermore, accounting for the pro-inflammatory properties of Bregs will allow for better optimization of transplant tolerance strategies through awareness of potential pro-inflammatory cytokine-induced complications post-transplant. This review examines the immune-suppressing and

-activating functions of the Breg-produced cytokine TNF-α and its synergistic role with IL-10, ultimately leading to transplantation tolerance. Using examples from outside the transplantation field, we also evaluate the translational potential of TNF-α therapies that had promising clinical outcomes in alleviating autoimmune-driven inflammation (11, 12). We finally discuss recent insights on the role of Bregs and TNF-α in transplantation to develop a more critical understanding of this unexpected Breg feature. Data analysis leads to a potential clinically applicable approach furthering the tolerogenic properties of TNF-α while suppressing inflammation through a selective TNF-α receptor blockade.

## Breg differentiation, function, and cytokine production

The suppressive functions of B cells and postulation on the existence of a subpopulation of regulatory B cells were first explored in the 1970s following observation by Ruth and Salvin on the suppression of delayed hypersensitivity reactions in guinea pigs after lymphoid cell transfer into antigen-sensitized recipients (13). The effort to characterize the molecular and biochemical mechanisms by which the regulatory subset of suppressive B cells was not made until the turn of the 21st century when three studies showed that B cells could suppress inflammation through IL-10 production in murine models of colitis, encephalomyelitis (EAE), and arthritis (2, 3, 14). These findings were extended to transplantation models with the observation that B cells were required to develop transplantation tolerance induced by antibody treatments (7, 15–17). Studies on genetically modified B cells and B cells from IL-10-deficient mice showed that defective Breg differentiation and function causes chronic inflammation (14). Bregs promote immunological tolerance through suppression of T cell expansion, downregulation of the production of pro-inflammatory cytokines, and maintenance of immune homeostasis through the synthesis of immune modulating cytokines TGF-β, IL-35, and IL-10 and by promotion of regulatory T cells (Tregs) (7, 14, 16, 18).

Transforming growth factor β (TGF-β) activates several pathways that regulate various cellular functions (19). Under standard physiological conditions, most B cells produce low levels of TGF-β, and regulation of TGF-β is dependent mainly on downstream signaling of multiple pathways in conjunction with the local cellular environment. TGF-β-producing B cells have been shown to regulate the immune response independent of IL-10 in various inflammatory diseases, including autoimmune diseases, cancer, and allergies (20). Experiments in mice have indicated that TGF-β-producing B cells trigger apoptosis of T and B cells and thereby prevent immune-mediated tissue destruction (21). Within the context of transplant tolerance, TGF-β plays potentially opposing roles by contributing to both long-term allograft tolerance and transplantation rejection in a concentration-dependent manner within the local environment (22, 23). At higher local concentrations, TGF-β signaling pathways increase Foxp3 expression in Tregs, which is crucial to the suppression of anti-graft responses and tolerance (22, 23). In contrast, when TGF-β is present at lower concentrations and exposed to specific pro-inflammatory cytokines such as IL-6 and IL-4, a synergistic effect occurs, promoting the differentiation of tissue-damaging Th17 and Th4 T cells (24). Interestingly, this provides insight into the importance of cytokine dosage and local environment when tailoring TGF-β in clinical settings.

IL-35 is another immunosuppressive and anti-inflammatory cytokine mainly secreted by regulatory T cells but is also expressed by Bregs (25). Bregs’ IL-35 receptor activation indirectly induces STAT1 and STAT3 gene activation, which contribute to the synthesis of proteins involved in multiple immune functions (26). Primarily, IL-35 induces the expansion of Treg and Breg subsets, thereby suppressing lymphocyte proliferation (27). Often considered the hallmark of functional Breg cells, IL-10 is perhaps the best-characterized cytokine associated with Breg suppressive activity. Its potent anti-inflammatory properties are central in limiting immune responses to pathogenic and foreign graft antigens. Its primary function is to tame inflammatory responses and inhibit T cell-mediated immune responses.

Upregulation of the immunoregulatory Nucleoside triphosphate diphosphohydrolases (NTPDase), CD39, and the transferrin receptor CD71 expression was found to be strongly associated with IL-10 expression by B cells (4). Other regulatory molecules, such as CD9, CD25, and CD197, also characterize IL-10-producing Bregs (4). Although the widespread dispersion of IL-10 Bregs in every Breg subset remains unclear, the IL-10 phenotype is a convenient surrogate marker for identifying Bregs. In transplantation, exogenous IL-10 has been found to prevent posttransplant ischemia-reperfusion injury and decrease acute rejection (18). IL-10-producing B cells have also been shown to be critical for preventing and controlling autoimmune disease and chronic inflammation (28). These IL-10 properties, which dampen immune responses to transplant and maintain immune homeostasis, support the use of IL-10-producing Bregs in treating and possibly preventing transplant rejection. Though commonly associated with these anti-inflammatory cytokines, Bregs are also now understood to produce pro-inflammatory cytokines. Indeed, many IL-10^+^ B cells co-express the pro-inflammatory cytokines IL-6 and TNF-α (4, 29). A similar result was found in a subset of Bregs termed Bregs-TLR, which are generated via activation of the TLR4 (LPS) and TLR9 (CpG) pathways (30).

One subpopulation of B cells, the CD25^+^CD39^hi^ B cells, are enriched within IL-10-producing B cells and are abundant among cells secreting TNF-α (4). These highly activated B cells demonstrate pro-inflammatory features while secreting anti-inflammatory immunoregulatory cytokines such as IL-10. The co-induction of IL-10 and TNF-α expression is corroborated in several studies (4, 29, 31–33). IL-10^+^ B cell populations enriched for higher protein-synthesis levels were phenotypically indistinguishable from TNF-α^+^ B cells. Among CD25^+^ and CD39^hi^ B cell populations, TNF-α^+^IL-10^+^ cells have been noted to be abundant (20% and 15%, respectively) (4). As approximately 30% of human peripheral blood and 60-80% of cord blood B cells express CD25, and >90% of all B cells in human peripheral blood are CD39^+^, TNF-α^+^IL-10^+^ cells may have a small but impactful role in human peripheral blood. The dynamics of IL-10 production coupled with pro-inflammatory TNF-α production by these B cell subsets should be given more attention in transplant immunology research.

## TNF-α function and signaling pathway

Tumor necrosis factor alpha (TNF-α) is a member of the TNF superfamily, which is involved in the regulation of immune cell functions but also in inducing fever, apoptotic cell death, muscle loss, inflammation, inhibition of tumorigenesis, and viral replication (34). Cytokines in the TNF superfamily are well-known in mammals for their crucial role in B cell differentiation, maturation, homeostasis, antigen presentation, and activation (34). TNF-α is a pleiotropic pro-inflammatory cytokine that regulates immune responses in health and disease via signaling pathways that control either cell survival or cell death. Mainly produced by activated macrophages, T cells, and NK cells TNF-α possesses both proliferative and cell death-inducing properties despite its narrow denomination as a necrosis factor (3). The implications of TNF-α in numerous biological processes are manifold and significant: TNF-α is involved in leukocyte trafficking and immune clearance, the promotion of dyslipidemia and insulin resistance, the formation of granulomas, and immune homeostasis (34).

**Figure 1:**
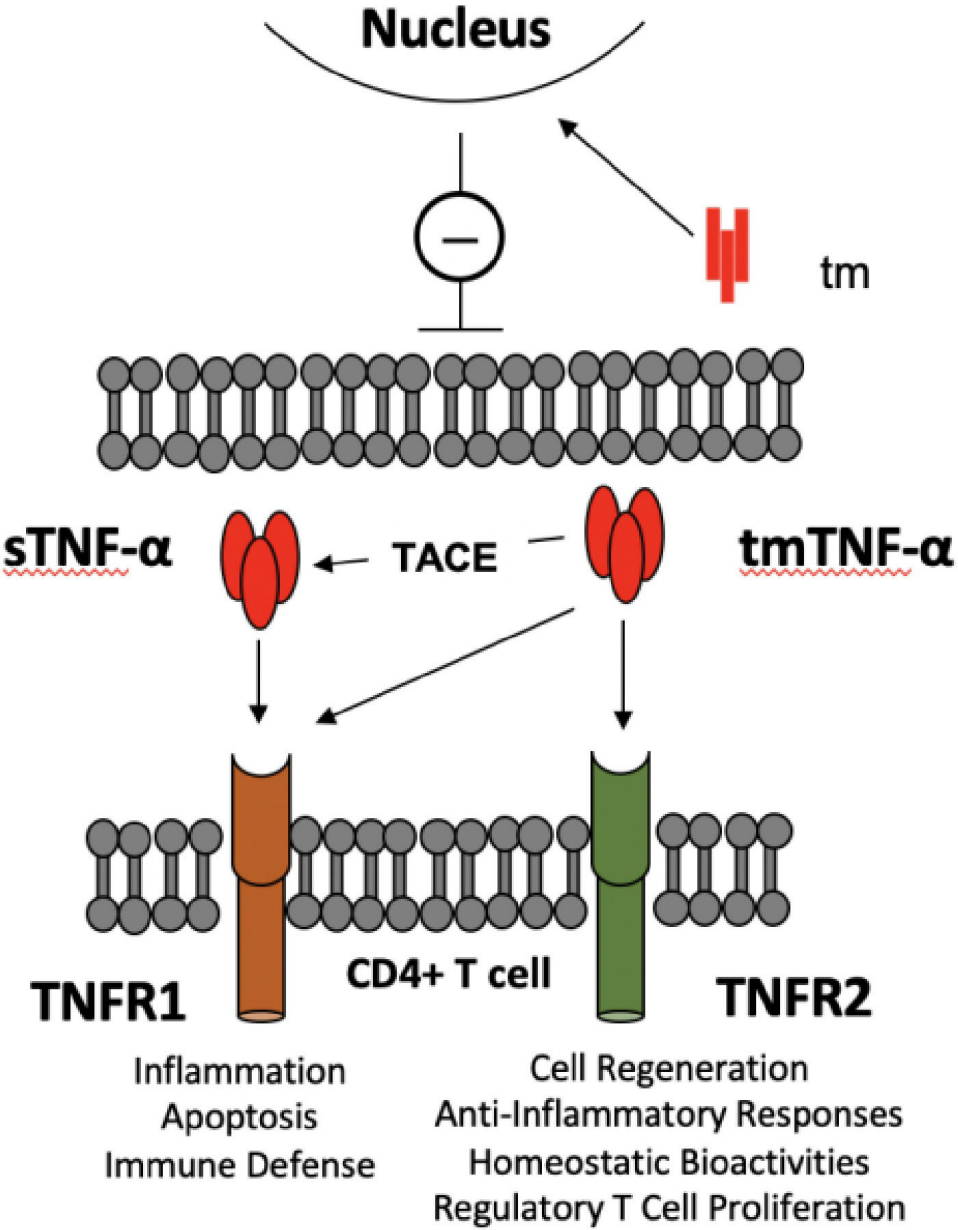
TNF-α -TNF-α receptors crosstalk between TLR-Bregs and effector CD4^+^ T cells. There are two molecular entities of TNF-α: a membrane-bound trimer of a single polypeptide chain (tmTNF-α), which is the precursor of a soluble trimer TNF-α (sTNF-α) obtained from cleavage by the tumor necrosis factor converting enzyme (TACE). The main causes of the pleiotropic effects of TNF-α are that sTNF-α selectively binds TNFR1 and generates pro-inflammatory activity. In contrast, tmTNF-α binds TNFR1 or R2, according to microenvironmental cues, to generate pro- or anti-inflammatory responses. In addition, the transmembrane domain (tm), resulting from the tmTNF-α cleavage, translocates to the nucleus, where it signals a pro-inflammatory response (8). The figure depicts the potential interactions in controlling CD4^+^ T cell activation by TNF-α producing TLR-Bregs. Because Tregs are TNFR1^low^, TNFR2^high^, TNFα produced by TLR-Bregs may also control the homeostasis of Treg cell numbers.

This cytokine appears in two molecular forms that each engage a different cell surface receptor. The soluble TNF-α (sTNF-α) binds to the type 1 receptor (TNFR1), whereas the membrane-bound form of TNF-α (tmTNF-α) is recognized by the type 2 receptor (TNFR2) (10, 11, 28). While TNFR1 is expressed ubiquitously on all cell types in the body, TNFR2 is mainly displayed on immune cells (10, 11, 28). Each ligand-receptor interaction results in receptor-specific cellular responses (10) (**Figure 1**). The engagement of TNFR1 triggers the formation of various signaling complexes that induce inflammatory responses, tissue or cell survival, and immune defense mechanisms. Activation of TNFR2, on the other hand, results in homeostatic bioactivities, like cell regeneration, proliferation, and survival (10). Ubiquitin plays a critical role in TNF-α signaling, enabling signal transduction complex formation and protein degradation. Ubiquitination indirectly modulates inflammatory responses and cell death signaling. Loss of function mutations of the ligase complexes that catalyze linear ubiquitination have been found in patients with significant immunodeficiencies and immune dysregulation (28).

Upon activation, B cells show a sequential pattern of cytokine expression. When stimulated by CpG, anti-CD40, IL-2, IL-21, and IL-35, B cells from human cultured PBMC expressed a first wave of pro-inflammatory cytokines TNF-α and IL-6, followed by IL-10. Indeed by 6 hours of cell culture most B cells expressed both TNF-α and IL-6, but less than 1% of them produced IL-10 (4). At 12-24 hours, B cells began to express both TNF-α and IL-10, and after 48 hours, TNF-α^+^ B cells substantially decreased in number, while the IL-10^+^ B cell population remained significant (4). This suggests that Bregs predominantly elicit pro-inflammatory signals in their early activation phase and subsequently develop into a more regulatory and anti-inflammatory cell subset in response to extrinsic cues. Further research is needed to understand the mechanism and reveal the molecular cues contributing to this cytokine shift.

## Blood IL-10:TNF-α ratio is a better indicator of clinical outcome than IL-10 levels alone

As the production of IL-10 characterizes the main subset of Breg cells involved in transplantation tolerance, one may evaluate the value of Bregs IL-10 as a marker of tolerance. Glass et al. addressed this issue in operationally tolerant liver allograft recipients (patients with stable graft function in the absence of immunosuppressive drugs). Breg involvement in tolerance was suggested by the fact that operationally graft-tolerant liver transplant recipients and transplant control patients had a significantly higher proportion of IL-10^+^ TNF-α^+^ B cells in stimulated PBMCs (16% and 18%, respectively), compared with control transplant recipients under maintenance immunosuppression (6% IL-10^+^ TNF-α^+^B cells) (4). There were no clear correlations between tolerance and the proportion of IL-10 only producing B cells, suggesting that this cell type was not the unique subset involved. On the other hand, the operationally tolerant cohort showed a roughly 2-fold higher log [IL-10^+^: TNF-α^+^] ratio than the control group, indicating that B cells producing high tolerogenic IL-10 and low graft-damaging TNF-α were significantly involved in operational tolerance (4).

The predictive value of a B cell IL-10:TNF-α ratio in graft acceptance was corroborated in other studies by Cherukuri et al., who showed that the ratio could measure cytokine polarization in B cell subsets and better indicate of regulatory function than IL-10 expression alone (29). Transitional B cells (TrBs), known as differentiation intermediates between immature bone marrow and peripheral mature B cells, exhibited the most anti-inflammatory cytokine profile *in vitro*. The study also found a reduced TrB IL-10:TNF-α ratio predictive of poorer clinical outcomes in allogeneic renal transplants (29). Furthermore, TrB cells taken from patients after graft rejection had lost their suppressive activities *in vitro* (29).

A study investigating blood cytokine levels of TNF-α, IL-1, IL-2, IL-6, IL-8, and IL-10 in cirrhotic patients undergoing orthotopic liver transplantation (OLT) determined that those who exhibited overexpression of both anti- and pro-inflammatory cytokines were still able to maintain a balanced inflammatory response to surgery (35). Surveys of IL-6:IL-10 and IL-10:TNF-α ratios from blood samples taken before, during, and after surgery indicated that IL-6:IL-10 was a better assessment of the inflammatory balance status after surgery while IL-10:TNF-α was a better predictor in septic patients.

The predictive value of the IL-10:TNF-α ratio has also been seen in fields other than that of transplantation. In a study of severe burn injury, IL-10:TNF-α plasma levels measured shortly after burn trauma were directly correlated with burn size, the severity of the injury, and the susceptibility to repeat infections (≥3) during recovery (31). In the study, patient burn size and injury severity were classified based on mean total body surface area (TBSA), inhalation injury, and full-thickness burn status. Cases falling below the cohort’s mean (≤ 41%) had a lower average IL-10:TNF-α ratio of 5.6. In comparison, those above the mean (≥ 42%) exhibited an average IL-10:TNF-α ratio of 19.2 (31). Additionally, when using the IL-10:TNF-α ratio as a predictor for susceptibility to repeat infections, patients less susceptible to repeat infections (≤ 2) exhibited an average IL-10:TNF-α ratio of 5.0. In contrast, patients susceptible to repeat infections (≥3) had an average IL-10:TNF-α ratio of 14.9. Establishing the IL-10:TNF-α ratio as a reliable biomarker for burn injury severity and predictor of susceptibility to repeat infections could be clinically valuable for identifying high-risk patient groups and implementing more personalized risk-stratified management of patients.

Collectively, these studies document the value of the IL-10:TNF-α ratio as a relevant indicator of an anti- to pro-inflammation shift and a potential predictor for clinical treatment outcomes. The significance of this metric has contrasting implications for the role of each cytokine, as it appears that a balance pro-inflammatory and anti-inflammatory cytokines are required for positive clinical results (4, 31, 35).

## TNF-α mediated inflammatory diseases

TNF-α plays a significant role in the pathogenesis of several inflammatory diseases, such as arthritis, rheumatoid arthritis, ulcerative colitis, and Crohn’s disease. In transplantation, TNF-α is integral to mediating the rejection or acceptance of a graft and is particularly well-documented in graft-versus-host disease (GvHD) (36–38). TNF-α is released from host cells during the initial onset of acute GvHD and enhances CD8^+^ T cell cytotoxic response (36), thereby intensifying the severity of the disease and compromising the pathways to graft immune tolerance. At present, recipients who develop acute GvHD are treated steroid based with immunosuppression in the form of steroids in the case of more severe symptoms or multi-organ involvement in the disease. Though anti-TNF-α antibody injections before transplantation of allogeneic bone marrow cells have been shown to reduce GvHD mortality in murine models, no selective therapy exists for patients with acute GvHD who do not respond to steroids (38).

In recent decades, kidney graft survival has significantly improved due to advances in the design and delivery of immunosuppressive drugs (39). However, the proportion of adverse effects caused by cancers and infections has now surpassed the rejection rate, likely due to over-immunosuppression (OIS) (39). The status of OIS is defined by the inability of the immune system to prevent adverse infections from pathogens such as Cytomegalovirus (CMV), Epstein-Barr virus (EBV), and Pneumocystis Jiroveci Pneumonia (PJP) (39). In a study by Bouchard-Bovin et al. (2019), PBMC samples from 73 kidney graft recipients were stimulated by EBV *in vitro* and analyzed for TNF-α-producing CD14^+^CD16^+^ cell populations (39). The criteria to be a patient with OIS required any combination of the following ≥3 times within 12 months: recurrence of infections, infection by opportunistic pathogens, or incidence of cancer. Notably, the number of TNF-α secreting CD14^+^CD16^+^ monocytes after EBV stimulation was reduced in patients falling into the OIS category. Conversely, there was no correlation between IOS patients and clinical measures of age, immunosuppressant levels/doses, renal functions, and time post-transplant (39). These findings indicate TNF-α as a potential biomarker for individualized immunosuppressive treatment of patients. However, studies on larger cohorts of patients will be required to substantiate these initial findings.

Recent studies have shed light on the mechanisms by which TNF-α fosters graft rejection. Jaber et al. (2020) performed syngeneic and allogeneic hepatocyte transplantation in dipeptidyl-peptidase-deficient (DPP) F344 rats, treated with mild lymphocyte immunosuppression (mofetil and tacrolimus) (40). As expected, syngeneic hepatocytes were successfully engrafted without immunosuppression, whereas allogeneic hepatocytes were rejected. The immunosuppressive treatment promoted the acceptance of both syngeneic and allogeneic cell grafts. An extensive analysis of cytokine, chemokine, and cognate receptor expression revealed that genes of TNF-α and its respective receptors maintained steady expression levels correlating with tolerance (40). Through the use of TNF-α antagonists, these studies also identified the CXCL-8 pathway, which promotes neutrophil recruitment and graft rejection, as a route by which the deleterious effects of TNF-α could be controlled.

## TNF-α inhibitors as a therapeutic agent

TNF-α has become increasingly recognized for its potent role in the pathogenesis of chronic inflammatory diseases leading to the emergence of various anti-TNF-α therapies that assert their effects through different pathways and mechanisms that should be understood as precautionary measures for potential complications when applied to other fields such as transplantation. When TNF-α is dysfunctional, diverse immunopathology can result, including rheumatoid arthritis, inflammatory bowel disease, Wegener granulomatosis, sarcoidosis, psoriasis, psoriatic arthritis, ankylosing spondylitis, juvenile chronic arthritis, atherosclerosis, and sepsis (9). Of the five TNF-α blocking agents that are licensed to treat TNF-α associated diseases, Infliximab (anti-TNF human-murine chimeric IgG1 monoclonal antibody), Adalimumab (human anti-human TNF-α antibody), Golimumab (human anti-TNF-α IgG1ϰ monoclonal antibody), and Certolizumab pegol (Fab’ fragment of humanized anti-TNF-α antibody) all trigger TGF-β production in human macrophages attributed to tmTNF-α reverse signaling (tmTNF-α acts as a receptor when interacting with TNFR2, anti-TNF antibody, or TNFR2-expressing cells leading to activation of intracellular signaling pathways) (9). The anti-TNF-α targeting molecules in Infliximab, Adalimumab, and Golimumab result in cell death via apoptosis, which is linked to TNF-α reverse signaling that triggers TGF-β production via activation of the MKK4 signaling pathway (9). TGF-β is known for its anti-inflammatory effects, potent in inhibiting neutrophil and T-cell adhesion to endothelial cells, down-regulating macrophages, and reducing the functions of TNF-α (9).

The clinical application of cytokine inhibitors, including those that antagonize the effects of TNF-α or IL-6, is often done with caution due to the complex nature of their signaling pathways, where alterations to one aspect may result in a myriad of unforeseen downstream effects such as infection or developing cancer (41, 42). The use of TNF-α inhibitors (monoclonal antibody Adalimumab, chimeric monoclonal antibody Infliximab, and TNF-α specific monoclonal antibody fragment Certolizumab pegol) as therapeutic agents for transplant recipients has recently been investigated for efficacy and safety in 6 renal transplant patients for treating ankylosing spondylitis (AS) (43). Disease activity was reduced with TNF-α inhibitors in all AS patients, as seen in Bath Ankylosing Spondylitis Disease Activity Index (BASDAI) scores shifting from high to moderate disease activity (43). In contrast, a study on 16 renal transplant recipients receiving anti-TNF-α treatment displayed an overall poor response indicated by adverse outcomes during treatment and subsequent improvement after cessation (41). While receiving treatment, half of the patients developed severe infections, four developed cancer, and two lost their grafts. This clinical study’s poor outcomes highlight the dangers of total TNF-α inhibition within the context of the pleiotropic activities of this cytokine.

Studies on the application of anti-TNF-α treatment in transplantation demonstrate the risks of using cytokine inhibitors to eliminate the function of cytokines that seem solely inflammatory yet are intricately intertwined in the signaling and function of complementary anti-inflammatory cytokines. Therapies have recently focused on targeting TNFR1 with antagonists and TNFR2 with agonists, which may alleviate the unwanted, severe side effects associated with anti-TNF-α treatment without disturbing the IL-10/TNF-α co-expression balance that contributes to a tolerogenic milieu (44).

## Therapeutic TNFR1 blockade and TNFR2 agonist for treatment of TNF-α mediated diseases

Increasing evidence supports TNFR2’s essential role in the proliferation and suppressive function of CD4^+^ Tregs, CD8^+^ Tregs, Myeloid-derived suppressor cells (MDSCs), and Bregs (45). In a study by Hijdra et al. (2012), sarcoidosis patients with either steady disease or experiencing remission of the autoimmune symptoms exhibited an increased percentage of CD4^+^TNFR2^+^ cells when compared to healthy individuals (45). This suggested the implication of TNFR2 signaling in down-regulating the immune system (45). As active TNFR2^+^ Treg cells surrounding tumors suppress anti-tumor CD8^+^ cytotoxic cells (46), cancer researchers have begun targeting this receptor with antagonistic therapies to inactivate Tregs and conversely foster cytotoxicity toward tumors. Because our research has shown that Tregs can be generated in the presence of Bregs-TLR, the development of which is also dependent upon TNFR2 signaling, we anticipate that future tolerance therapies may have to maintain or even amplify TNFR2 signaling (30).

Although it is known that TNF-α disrupts both differentiation and function of TGF-β-induced Tregs in autoimmune diseases via the Akt and Smad3 signaling pathways, TNF-α has also been shown to expand murine natural Tregs both *in vivo* and *in vitro* (47). Furthermore, in murine models, our TLR-Bregs induce CD4^+^CD25^+^FoxP3^+^ Tregs *in vivo* in a TGF-β dependent manner; however, identification of the specific Treg subpopulations requires further research (30). A study carried out by Yang et al. looked at the effects of TNF-α on two subsets of Tregs: induced Tregs (iTregs) from CD4^+^CD25^-^ in the periphery or naive CD4^+^ cells *in vitro* and natural Tregs (nTregs) originating from the thymus. The study found that TNF-α exposure to TNFR1^-/-^ mice promoted naïve CD4^+^ T cells to differentiate into iTregs while TNFR2^-/-^ mice iTreg induction was hampered indicating TNF-α’s ability to induce Tregs and the important role of TNFR2 in Treg induction.

To demonstrate the dichotomous relationship between TNFR1 and TNFR2 function, Richter et al. (2021) used an experimental autoimmune EAE animal model of multiple sclerosis to show that mice deficient in TNFR1 developed a much less severe form of the disease. In contrast, mice without TNFR2 had significantly more severe signs of EAE disease (11). Beyond its implications in understanding the antithetical functions of TNFR1 and TNFR2, this study further demonstrates the need for more advanced treatments that prevent potentially counterproductive anti-TNF therapeutics and increase positive clinical outcomes reliant on TNFR2 mechanisms.

Since TNFR1 is thought to be the primary promoter of inflammation and cell death in the TNF signaling pathway, it may be possible to use a therapeutic blockade of TNFR1 signaling while keeping homeostatic TNFR2 signaling intact as a treatment for TNF-mediated diseases (48, 49). This may be achieved at the level of ligand or receptor, though most approved TNFR1 blockade therapies to date interfere with TNF-α proinflammatory function at the level of the TNF ligand (44). As opposed to broad anti-TNF-α treatment that globally inhibits binding of TNF to both TNFR1 and TNFR2 receptors, selective inhibition of TNFR1 is achieved through receptor-specific modified ligands or monoclonal antibodies (44).

Clinical research in TNF-mediated disease where complete TNF inhibition is contradictory and has shown to have severe unwanted side effects is required to fully confirm the therapeutic benefits of TNFR1 blockade fully. Preclinical studies using dominant-negative inhibitors of soluble TNF (DN-TNF) to block soluble TNF binding to TNFR1 while sparing the binding of transmembrane TNF to TNFR2 have shown promising results (11, 44).

Patients receiving allogeneic hematopoietic cell transplantation (HCT) often have GvHD maintained largely by TNF-α release (50). Increases in plasma TNFR1 levels are expected in patients experiencing GvHD. They correlate with increased TNF-α levels that are directly harmful to the target organ and increase the donor’s immune responses to host tissues (50). However, GvHD treatment options using TNF-α blockade, such as Infliximab which not only neutralizes TNF-α but also induces cell death of TNF-α producing cells, should be carried out with caution. Indeed, similar to the anti-TNF-α renal transplant study, a study on 11 patients suffering from acute GI GvHD treated with Infliximab delivered poor results: only two patients survived (33, 36). In another study by Choi et al. (2008), patients with GvHD had TNFR1 plasma levels nearly doubled by day 7 after HCT, in correlation with tissue damage (50). The link between TNFR1 plasma levels and GvHD emphasizes its importance as a predictor for GvHD. It further supports research targeting this receptor as a potential treatment of TNF-α mediated diseases. As noted above, caution should underline this approach, given the complex nature of TNFR1/TNFR2 signaling.

A more selective TNFR1 antagonist was developed by Richter et al. (2021) as a modified anti-TNFR1 antibody, Atrosimab. This molecule can selectively block TNFR1 signaling while leaving the anti-inflammatory TNFR2 pathways unaffected. Its therapeutic efficacy was demonstrated in murine models of acute and chronic inflammation, including non-alcoholic steatohepatitis (NASH) and EAE (11). Use of Atrosimab *in vivo* was also shown to inhibit TNF-induced release of inflammatory cytokine IL-6, as well as TNF-induced weight loss. In a murine model of rheumatoid arthritis, low-dose Atrosimab treatment slowed disease progression, and high-dose treatment improved therapeutic activity (11). Though the efficacy of Atrosimab in promoting transplant tolerance is unknown, its success in treating other chronic inflammatory diseases makes it a promising prospect as a therapeutic.

By contrast, TNFR2 signaling has been shown to improve peripheral regulatory T cell (Treg) expansion and sustain cell survival, which can be leveraged to promote tolerance of allogeneic transplants and protect against GvHD (51). As discussed above, treatments involving generic TNF blockers like Infliximab worsened chronic autoimmune disease or GvHD symptoms (12, 50). Alternately, therapeutic approaches using selective agonists of TNFR2 have boosted Treg expansion and reduced GvHD symptoms in patients who are recipients of allogeneic bone marrow cells (52). These encouraging findings are supported by related murine models using TNFR2 antagonists in tumor models that have shown opposite outcomes: decrease of Treg activity and concomitant activation and expansion of anti-tumor CD4^+^ and CD8^+^ T cells (46). The *in vivo* applications of TNFR2 agonists have also shown promising outcomes in tumor treatments (53, 54). Among the agonistic antibodies showing success in reducing tumors in mouse models, the agonistic antibody Y9 mediated its effects through IFNγ^+^CD8^+^ T cells and NK cells. The Y9 antibody treatment increased the proliferation of CD8^+^, IFN-γ^+^ T cells, and the production of cytokines but did not deplete Treg cells. Treatment with Y9 alone resulted in an anti-tumor response more potent than anti-PD-1 alone. Combined, these two antibodies showed synergistic effects on improved anti-tumor responses and increased survival rates (53, 54). Another potentially therapeutic TNFR2 agonist termed TNFR2-selective agonist TNF mutant R2agoTNF was created using a proprietary cytokine modification technique based on macrophage display. Alone, the R2agoTNF mutant initiates cell signaling through TNFR2 and induces proliferation of CD4^+^CD25^+^ Tregs *in vitro*, but internal cross-linking by IgG-Fc fusion allowed for a similar effect *in vivo* (55).

## Concluding remarks

Here we discuss the role TNF-α plays in the pathogenesis of many autoimmune diseases and their relation to Breg suppressive function with the ultimate goal of designing a combinatorial therapy involving blocking the TNFR1 receptor associated with inflammatory TNF-α signaling, activation of TNFR2 signaling involved with an anti-inflammatory response, and TLR-Bregs to suppress effector T cell proliferation and induce regulatory T cells. Regulatory B cells are primarily known for their ability to suppress inflammation, promote immunological tolerance, and downregulate pro-inflammatory cytokine production via the immune modulating cytokines IL-10, TGF-β, and IL-35. We highlight studies detailing the expression of TNF-α in IL-10 producing B cells that initially appears antithetical to their function, but after further investigation of TNF-α binding dynamics to its two receptors (TNFR1 and TNFR2) that allow for its pleiotropic, opposing signaling pathways, the potential inhibition of the inflammatory pathway while maintaining the homeostatic signaling of the other becomes an apparent solution to many of the autoimmune diseases mediated by TNF-α dysregulation. Furthermore, we discuss the clinical applications of the IL-10:TNF-α ratio, using it as an predictor of pro- and anti-inflammatory treatment outcomes in autoimmune diseases and transplantation.

**Figure 2:**
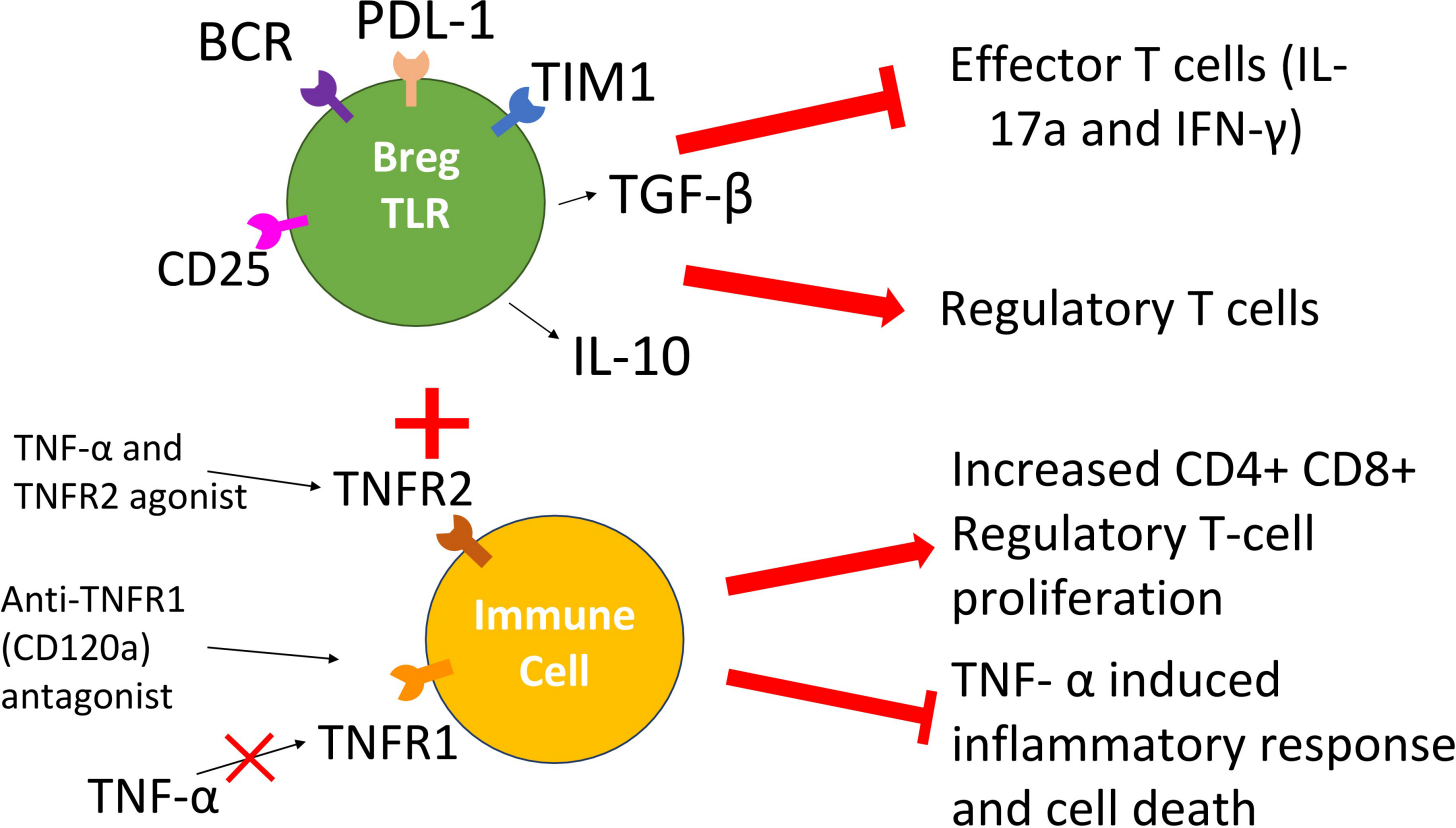
Scheme of the proposed combinatorial Breg-TLR/TNFR1/R2 therapy. The approach involves the combined administration of Bregs-TLR cells that induce suppressive Treg cells (suppression of anti-graft responses), a TNFR1 antagonistic antibody (reduction of inflammatory responses toward the transplant) and a TNFR2 agonistic drug (boost of tolerogenic responses).

The above studies on TNFR1 and TNFR2 antagonistic and agonistic treatments call attention to a novel tolerance-inducing approach that would combine the administration of Breg-TLR cells (to stimulate Treg emergence and function) with either TNFR1 antagonists (to block pro-inflammatory responses of TNF-α), TNFR2 agonists (to stimulate tolerogenic responses of TNF-α), or both (**Figure 2**). Results from several experimental and clinical studies support the proposed strategy. Our work on a subset of Bregs, Bregs-TLR, suggest they suppress *in vivo* graft-specific T cell proliferation while inducing Tregs via the TGF-β pathway (30). A TNFR1 antagonistic CD120a antibody such as Atrosimab has not been tested within a transplant environment; however, its ability to inhibit the inflammatory response associated with downstream TNFR1 signaling demonstrated in autoimmune diseases makes it an interesting prospect for the proposed combinatorial therapy. Lastly, a TNFR2 agonist like R2agoTNF that induces the *in vitro* proliferation of CD4^+^CD25^+^ Tregs could play a decisive role in preventing the unwanted immune response associated with transplant rejection. A combination therapy that involves treatments exerting the effects above could provide improved transplant outcomes reliant on the gestalt of treatments rather than the administration of the individual components alone.

## Author contributions

SP and MY generated the figure, wrote, and edited manuscript. KD, and QF provided critical reading and edited of the manuscript CL and JM provided critical reading, edited, and wrote the manuscript. All authors contributed to the article and approved the submitted version.
